# A Pepducin Derived from the Third Intracellular Loop of FPR2 Is a Partial Agonist for Direct Activation of This Receptor in Neutrophils But a Full Agonist for Cross-Talk Triggered Reactivation of FPR2

**DOI:** 10.1371/journal.pone.0109516

**Published:** 2014-10-10

**Authors:** Michael Gabl, Malene Winther, Sarah Line Skovbakke, Johan Bylund, Claes Dahlgren, Huamei Forsman

**Affiliations:** 1 Department of Rheumatology and Inflammation Research, University of Gothenburg, Gothenburg, Sweden; 2 Department of Drug Design and Pharmacology, Faculty of Health and Medical Sciences, University of Copenhagen, Copenhagen, Denmark; Medical School of Hannover, Germany

## Abstract

We recently described a novel receptor cross-talk mechanism in neutrophils, unique in that the signals generated by the PAF receptor (PAFR) and the ATP receptor (P2Y_2_R) transfer formyl peptide receptor 1 (FPR1) from a desensitized (non-signaling) state back to an actively signaling state (Forsman H *et al.*, PLoS One, 8:e60169, 2013; Önnheim K, *et al.*, Exp Cell Res, 323∶209, 2014). In addition to the G-protein coupled FPR1, neutrophils also express the closely related receptor FPR2. In this study we used an FPR2 specific pepducin, proposed to work as an allosteric modulator at the cytosolic signaling interface, to determine whether the cross-talk pathway is utilized also by FPR2. The pepducin used contains a fatty acid linked to a peptide sequence derived from the third intracellular loop of FPR2, and it activates as well as desensensitizes this receptor. We now show that neutrophils desensitized with the FPR2-specific pepducin display increased cellular responses to stimulation with PAF or ATP. The secondary PAF/ATP induced response was sensitive to FPR2-specific inhibitors, disclosing a receptor cross-talk mechanism underlying FPR2 reactivation. The pepducin induced an activity in naïve cells similar to that of a conventional FPR2 agonist, but with lower potency (partial efficacy), meaning that the pepducin is a partial agonist. The PAF- or ATP-induced reactivation was, however, much more pronounced when neutrophils had been desensitized to the pepducin as compared to cells desensitized to conventional agonists. The pepducin should thus in this respect be classified as a full agonist. In summary, we demonstrate that desensitized FPR2 can be transferred back to an actively signaling state by receptor cross-talk signals generated through PAFR and P2Y_2_R, and the difference in agonist potency with respect to pepducin-induced direct receptor activation and cross-talk reactivation of FPR2 puts the concept of functional selectivity in focus.

## Introduction

Human neutrophils, the most prominent effector cells in innate immune reactions and inflammation, express a number of different chemoattractant receptors including the receptors for the complement component C5a (C5aR), the leukotriene LTB_4_ (BLT1), the chemokine IL-8 (CXCR1 and CXCR2), the platelet activating factor (PAFR), the nucleotide ATP (P2Y_2_R), and two members of the formyl peptide receptor family (FPR1 and FPR2) [1], [2], [3]. All these receptors belong to the family of seven transmembrane G protein-coupled receptors (GPCRs), a large and diverse group of cell surface receptors important for many cellular activities in health and disease [4], [5], [6], [7]. The precise mechanisms that regulate neutrophil functions through this group of pertussis toxin sensitive GPCRs is not known in detail, but the paradigm for how cellular responses are triggered is in agreement with the generally accepted GPCR-signaling scheme [8]. In this scheme, the starting point is ligand binding, and conventional GPCR agonists do not cross the plasma membrane barrier but bind to domains of their cognate receptor exposed on the extracellular side of the cell membrane and/or to transmembrane receptor parts localized close to the cell surface. The agonist occupied receptors are stabilized in an active signaling conformation that transfers the primary signal to the G-protein binding structures present in the cytosolic domains of the receptor and induces a receptor driven dissociation of the heterotrimeric G-protein complex into actively signaling subunits [2], [8], [9]. Subsequently, signaling is terminated (or directed towards endocytic uptake of the receptor-ligand complex) and the occupied receptor becomes refractory to further stimulation with the same agonist and to other agonists that bind the same receptor, an effect commonly termed homologous receptor desensitization [10], [11].

The non-signaling conformation of the desensitized receptor has for long been regarded as a “state of no return” in the sense that no signaling activity can be induced by this receptor unless it is first endocytosed, freed of agonist in lysosomal compartments and recycled back to the plasma membrane. The classical mode of receptor activation described above has recently been challenged as it has been shown, on the one hand that desensitized neutrophil receptors can be reactivated through a novel receptor cross-talk mechanism [12], [13] and, on the other hand that receptors can be activated/inhibited by allosteric modulators without a direct interaction with the surface exposed binding sites used by conventional agonists [14], [15]. Pepducins are one group of such allosteric modulators, and they all contain a fatty acid (usually a palmitoyl group) linked to a peptide sequence identical to one of the intracellular loops (typically the third) or the cytoplasmic tail of the receptor to be targeted [16], [17]. The “allosteric modulation” mechanism by which pepducins can activate or inhibit G-protein signaling is not yet understood [17], but it is proposed to involve an interaction with the signaling interface of targeted receptors from the cytosolic side. The discovery of pepducins has generated new insights into GPCR signaling, and therapeutical potential in disease models has been documented [18], [19]. Receptor selective pepducins have been identified for many GPCRs and a neutrophil activating pepducin was recently added [14] to the large number of different substances (agonists, antagonists and inhibitors of signaling) that have been described to affect FPR2 mediated functions in these cells [1], [2]. The peptide sequence of this FPR2 specific pepducin (F2Pal_16_) was identical to the third intracellular loop of FPR2, and subsequently a shorter variant (F2Pal_10_) was shown to be more potent than the “full-length” pepducin [14]. This shorter variant is supposed to interact allosterically with FPR2 from the cytosolic side after passage across the plasma membrane [14], [17].

In neutrophils, co-expressed GPCRs have the ability to communicate with one another and there is a pronounced hierarchy between the different receptors. Given that multiple inflammatory mediators recognized by neutrophil GPCRs are present simultaneously at sites of inflammation, the outcome of a neutrophil response is likely to be regulated by so-called hierarchical receptor cross-talk to ensure that cells can migrate directionally also in opposing gradients of chemoattractants [20]. Such a cross-talk whereby hierarchically strong (end-point) chemoattractants overrule weaker chemoattractants is mediated by heterologous receptor desensitization, meaning that ligation and activation of one (hierarchically strong) receptor may desensitize also non-occupied but hierarchically weaker receptors of other ligand specificities [20]. For example, FPR ligands desensitize cells in response not only to FPR agonists, but also to IL-8 and LTB_4_, binding to CXCR1/2 and the BLT1, respectively. No desensitization is, however, obtained when the agonist order is reversed [20]. We recently described a novel receptor cross-talk mechanism in neutrophils, unique in that the signals generated by the ATP receptor (P2Y_2_R) [12] and the PAF receptor (PAFR) [13] and transfer FPR1 from a desensitized (non-signaling) state back to an actively signaling state, resulting in a primed PAF or ATP response. To our knowledge, this is a unique cross-talk mechanism between two GPCRs, although the precise signaling pathway involved still needs further investigation.

In this study, we explored the reactivation potential of desensitized FPR2 through receptor cross-talk with PAFR and P2Y_2_R. Although the pepducin F2Pal_10_ was less potent than the conventional FPR2 peptide agonist WKYMVM in direct receptor activation determined as neutrophil NADPH-oxidase activity, cells desensitized to the pepducin were hyper-active when stimulated with PAF or ATP and the PAF- as well as the ATP-induced activity in pepducin desensitized cells was substantially inhibited by an FPR2 specific inhibitor, showing that activation is achieved through the recently described receptor cross-talk signaling route that transfers desensitized FPRs back to a signaling state.

## Materials and Methods

### Ethics Statement

This study, conducted at the Sahlgrenska Academy in Sweden, includes blood from buffy coats obtained from the blood bank at Sahlgrenska University Hospital, Gothenburg, Sweden. According to the Swedish legislation section code 4§ 3p SFS 2003∶460 (Lag om etikprövning av forskning som avser människor), no ethical approval was needed since the buffy coats were provided anonymously and could not be traced back to a specific donar.

### Chemicals

The hexapeptide WKYMVM, and the PIP_2_-binding peptide PBP10 (for details about this inhibitory molecule see [21]) as well as the pepducins were synthesized and HPLC-purified by CASLO Laboratory (Lyngby, Denmark). The pepducins were synthesized by Fmoc solid phase peptide synthesis and N-terminal palmitoylation was made on the resin as the last step before deprotection of side chains. Peptides were purified by HPLC on a C18 column and the correct sequence of each peptide was verified by MALDI-TOF Mass Spectrometry. The FPR2 antagonist WRW_4_ was from Genscript Corporation (Scotch Plains, NJ, USA). Isoluminol, latrunculin A, TNFα, Pertussis toxin and ATPγS were obtained from Sigma (Sigma Chemical Co., St. Louis, MO, USA). Cyclosporin H was kindly provided by Novartis Pharma (Basel, Switzerland) and PAF was from Avanti Polar Lipids Inc. (Alabama, USA). Peptides were dissolved in DMSO and stored at −70°C until use. Subsequent dilutions of all reagents were made in Krebs-Ringer phosphate buffer (KRG, pH 7.3; 120 mM NaCl, 5 mM KCl, 1.7 mM KH_2_PO_4_, 8.3 mM NaH_2_PO_4_ and 10 mM glucose) supplemented with Ca^2+^ (1 mM) and Mg^2+^ (1.5 mM). The PAFR antagonist WEB2086 was from Tocris Bioscience (Bristol, UK). Dextran and Ficoll-Paque were obtained from GE-Healthcare Bio-Science (Uppsala, Sweden). Horseradish peroxidase (HRP) was obtained from Boehringer Mannheim (Germany). Calyculin A was purchased from Nordic Biosite (Sweden). The Fura-2, Fura-Red and Fluo3 were from Life Technologies Europe (Stockholm, Sweden). Kinase inhibitors were obtained from Calbiochem (LaJolla, CA, USA).

The competitive P2Y_2_R specific antagonist AR-C118925 [22], synthesized according to the structure presented at the American Chemical Society [23] following the procedures laid down in a patent [24], was a generous gift of Dr Christa Müller, University of Bonn, Germany.

### Isolation of human neutrophils

Human peripheral blood neutrophils were isolated from buffy coats from healthy blood donors using dextran sedimentation and Ficoll-Paque gradient centrifugation as described [25]. The remaining erythrocytes were disrupted by hypotonic lysis, the neutrophils were washed twice, resuspended in KRG, and stored on melting ice until use. This isolation procedure permits cells to be purified with minimal granule mobilization.

### Neutrophil NADPH-oxidase activity

The NADPH-oxidase activity was determined using isoluminol-enhanced chemiluminescence (CL) [26], [27]. The CL activity was measured in a six-channel Biolumat LB 9505 (Berthold Co., Wildbad, Germany), using disposable 4-ml polypropylene tubes with a 900 µl reaction mixture containing 10^5^ cells, isoluminol (2×10^−5^ M) and HRP (2 U). The tubes were equilibrated in the Biolumat for 5 min at 37°C, after which the stimulus (100 µl) was added and the light emission was recorded continuously. Receptor desensitized cells were defined as naïve (non-desensitized) cells that had first been stimulated with a receptor specific agonist and returned to baseline after the resulting release of superoxide before a second stimulation. When reactivation experiments were performed with antagonists, the antagonists were added to the CL reaction mixture 1 min before the second stimulation. For studies with signaling inhibitors, cells were incubated with inhibitors at 37°C for 15 min before agonist addition. Control cells received no treatment but were incubated under the same conditions.

### Expression of formyl peptide receptors in HL-60 cells

The procedures used to obtain stable expression of FPR1 and FPR2 in undifferentiated HL-60 cells have been previously described [28]. To prevent possible auto-differentiation due to the accumulation of differentiation factors in the culture medium, cells were passed twice a week before they reached a density of 2×10^6^ cells/ml. At each passage, an aliquot of the cell culture was centrifuged, the supernatant was discarded and the cell pellet was resuspended in fresh RPMI 1640 medium containing FCS (10%), PEST (1%), and G418 (1 mg/ml).

### Calcium mobilization

Neutrophils or transfected HL-60 cells at a density of 5×10^7^ cells/ml in KRG containing 0.1% BSA were loaded with 5 µM FURA 2-AM for 30 minutes in the dark at room temperature. The cells were then diluted 1/2 in RPMI 1640 culture medium without phenol red (PAA Laboratories GmbH, Pasching, Austria) and centrifuged. Finally, the cells were washed once with KRG and resuspended in the same buffer at a density of 2×10^7^/ml. Calcium measurements were carried out in a PerkinElmer fluorescence spectrophotometer (LC50), with excitation wavelengths of 340 nm and 380 nm, an emission wavelength of 509 nm, and slit widths of 5 nm and 10 nm, respectively. The transient rise in intracellular calcium is presented as the ratio of fluorescence intensities (340 nm: 380 nm) detected. The measuring cuvette contained catalase (2000 U) to counteract inactivation of the chemoattractants by the MPO-H_2_O_2_-system [29]. The transient rise in intracellular calcium was also examined using an Accuri C6 flow cytometer (Becton Dickinson Sparks, MD, USA) with cells loaded with Fluo-3 AM (4 µg/ml) and Fura Red AM (10 µg/ml). The relative calcium level was expressed as a ratio between Fluo-3 and Fura Red (FL-1/FL-3) fluorescence over time.

### Data analysis

Data analysis was performed using GraphPad Prism 6.0 (Graphpad Software, San Diego, CA, USA).

## Results

### The FPR2 derived pepducin F2Pal_10_ activates FPR2 but not the closely related FPR1

**Figure 1:**
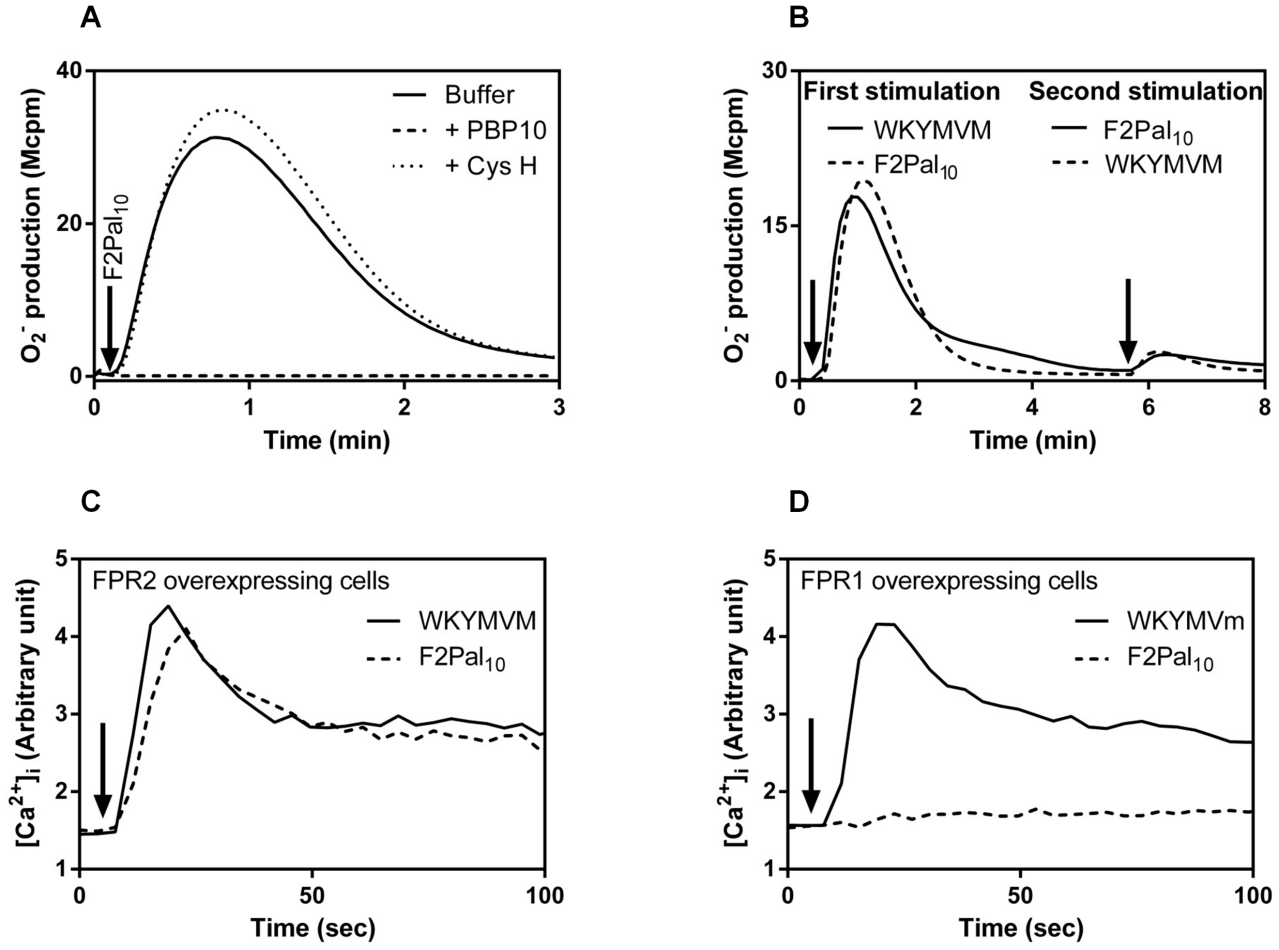
The F2Pal_10_ pepducin specifically activates FPR2. **A.** Primary human neutrophils were activated by F2Pal_10_ (500 nM) in the absence (solid line) or presence of the receptor specific inhibitor PBP10 (FPR2 specific inhibitor; 1 µM final concentration, dashed line) or the FPR1 antagonist cyclosporin H (CysH; 1 µM final concentration, dotted line) and the release of superoxide anions was recorded continuously. A representative experiment out of more than five is shown and the time point for F2Pal_10_ addition is indicated by an arrow. Abscissa: Time of study (min); ordinate: Superoxide production (10^6^× counts per minute; Mcpm). **B.** F2Pal_10_ desensitizes neutrophils in their response to the conventional FPR2 agonist WKYMVM. Primary human neutrophils were first activated by WKYMVM (40 nM; time point for addition is indicated by the first arrow, solid line) or F2Pal_10_ (500 nM; time point for addition is indicated by the first arrow, dashed line) and five minutes later reactivated by F2Pal_10_ (500 nM; time point for addition is indicated by the second arrow, solid line) or WKYMVM (40 nM; time point for addition is indicated by the second arrow, dashed line). The release of superoxide anions was recorded continuously. A representative experiment is shown. Abscissa: Time of study (min); ordinate: Superoxide production (Mcpm). **C and D.** The F2Pal_10_ pepducin triggers an increase in intracellular calcium when added to undifferentiated HL-60 cells overexpressing FPR2. The F2Pal_10_ pepducin (broken lines; 10 nM final concentration) was added to Fura-2 labeled FPR2 overexpressing cells (**C**) or FPR1 overexpressing cells (**D**), and the concentration of free cytosolic calcium was monitored by the Fura-2 fluorescence. Representative calcium responses induced by control peptides (WKYMVM for FPR2 and WKYMVm for FPR1) are included (solid lines) for comparison. Abscissa, time of study (sec); ordinate, fluorescence (arbitrary units).

A lipopeptide/pepducin with an amino acid sequence identical to the entire third intracellular loop of FPR2 was recently demonstrated to activate FPR2 [14], [30]. Also a shorter variant of this pepducin, F2Pal_10_, with a peptide sequence spanning amino acid 227 to 236 (KIHKKGMIKS) of the third intracellular loop of FPR2 activated neutrophils to produce superoxide (Fig 1A). The response was completely abolished by the FPR2 selective inhibitor PBP10 or the WRW_4_ peptide antagonist but it was not affected by cyclosporin H, a reversed agonist that specifically inhibits the closely related FPR1 (shown for PBP10 and cyclosporin H in Fig 1A). We conclude from these data that the neutrophil response induced by F2Pal_10_ is mediated through FPR2, and although the third intrallular loop of FPR2 differs in only two amino acids from the corresponding part of FPR1, the pepducin did not cross-react with FPR1. The receptor preference for FPR2 was further supported by desensitization data showing that neutrophils pre-activated with F2Pal_10_ were non-responsive to a second stimulation with the established FPR2 specific peptide agonist WKYMVM and vice versa (Fig 1B). The receptor preference of the F2Pal_10_ pepducin was confirmed using transfected cells over-expressing one of the FPRs. Undifferentiated HL-60 cells expressing FPR2 responded with a robust and transient increase in intracellular calcium upon challenge with F2Pal_10_ and the time course of the response was very similar to that induced by WKYMVM (Fig 1C). HL-60 cells expressing FPR1 were unresponsive, as no transient rise in intracellular Ca^2+^ was induced by F2Pal_10_. These cells were fully responsive to a control peptide (Fig 1D), which activates both FPR1 and FPR2. In summary, the FPR2 pepducin F2Pal_10_ is an FPR2 specific agonist that activates neutrophils to produce superoxide anions and the Ca^2+^ data presented illustrate the receptor specificity in that the correct receptor (in this case FPR2) has to be expressed in order for the pepducin to mediate its effect.

### The F2Pal_10_ pepducin is a partial FPR2 agonist for direct receptor-mediated NADPH-oxidase activation

**Figure 2:**
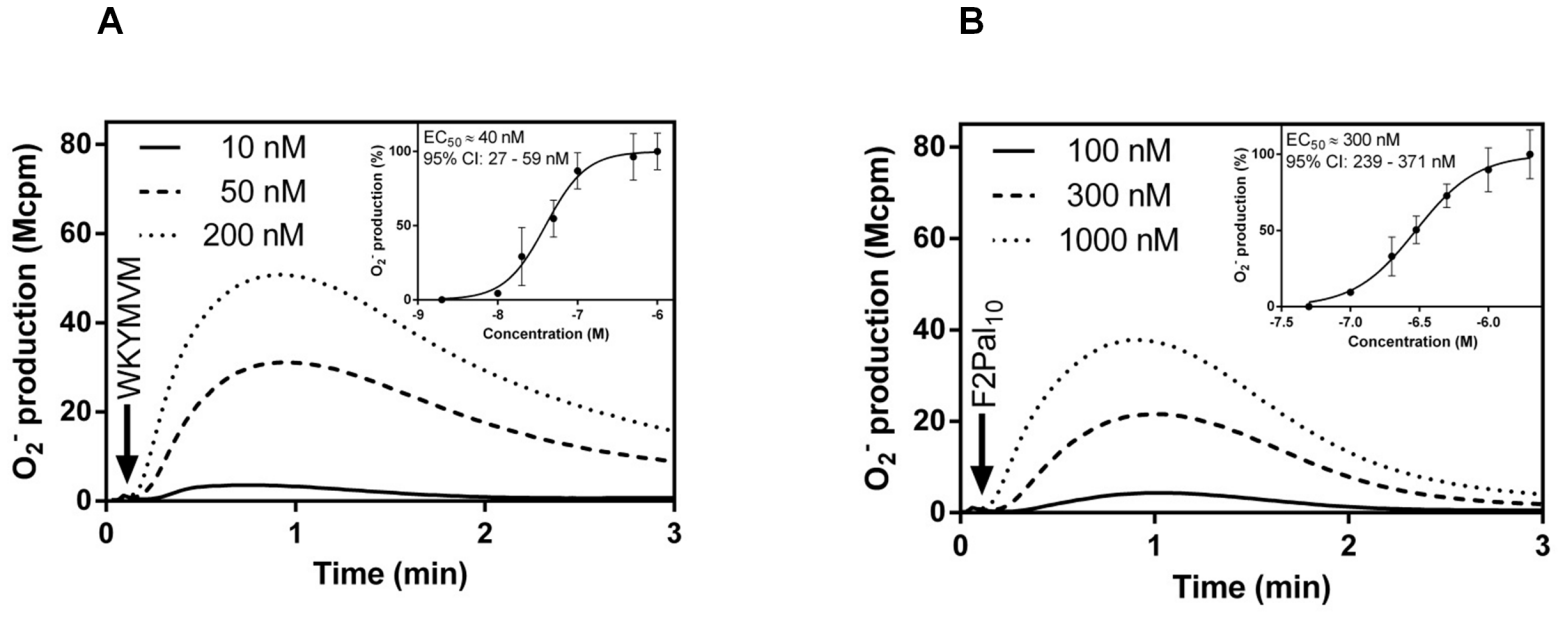
The F2Pal_10_ pepducin is a partial agonist in triggering superoxide release from human neutrophils. Human neutrophils were activated by different concentrations of WKYMVM (10–1000 nM; **A**) or F2Pal_10_ (50–2000 nM; **B**) and the release of superoxide anions was recorded continuously. Representative experiments out of more than five are shown and an arrow indicates the time point for agonist addition. Abscissa: Time of study (min); ordinate: Superoxide production (Mcpm). The dose responses for WKYMVM (**A**, inset) and F2Pal_10_ (**B**, inset) from three independent experiments are summarized in the insets, data are presented as normalized peak response with the fitted curves.

A full agonist has a high efficacy and induces a full cellular response when added in sufficient concentrations, whereas a partial agonist has lower efficacy and produces a sub-maximum response even when high concentrations are applied. We show that the conventional peptide agonist WKYMVM and the F2Pal_10_ pepducin interact with FPR2 and that the downstream signaling cascade leads to an activation of the superoxide anion producing NADPH-oxidase (Fig 2). Moreover, neutrophils challenged with the FPR2 specific peptide agonist WKYMVM produced and released superoxide anions in a dose-dependent manner with an EC_50_ concentration of around 40 nM (Fig 2A). A response with very similar kinetics was induced by the F2Pal_10_ pepducin with an EC_50_ value of around 300 nM (Fig 2B). Higher concentrations of F2Pal_10_ were required to reach maximum levels of superoxide release and no significant increase was obtained with pepducin concentrations above 500 nM (Fig 2B). Further, the maximum response induced by F2Pal_10_ reached only 70% of that induced by WKYMVM (Fig 2).

Based on the lower efficacy of the F2Pal_10_ pepducin in direct receptor mediated activation of the neutrophils compared to WKYMVM, we conclude that the pepducin acts as a partial agonist. The fact that F2Pal_10_ competes with the FPR2 specific peptide WKYMVM, as demonstrated in a binding assay using a fluorescent labeled peptide ([14], Fig S1), suggests that the amount of superoxide anion induced by a saturating concentration of WKYMVM (1 µM) should be higher than the response induced by the combined action of WKYMVM (1 µM) and F2Pal_10_ (1 µM). This was also the case (Fig S2), supporting the notion that the F2Pal_10_ pepducin is a partial agonist.

### Cross-talk signals generated by the PAFR/P2Y_2_R reactivate desensitized FPR2

**Figure 3:**
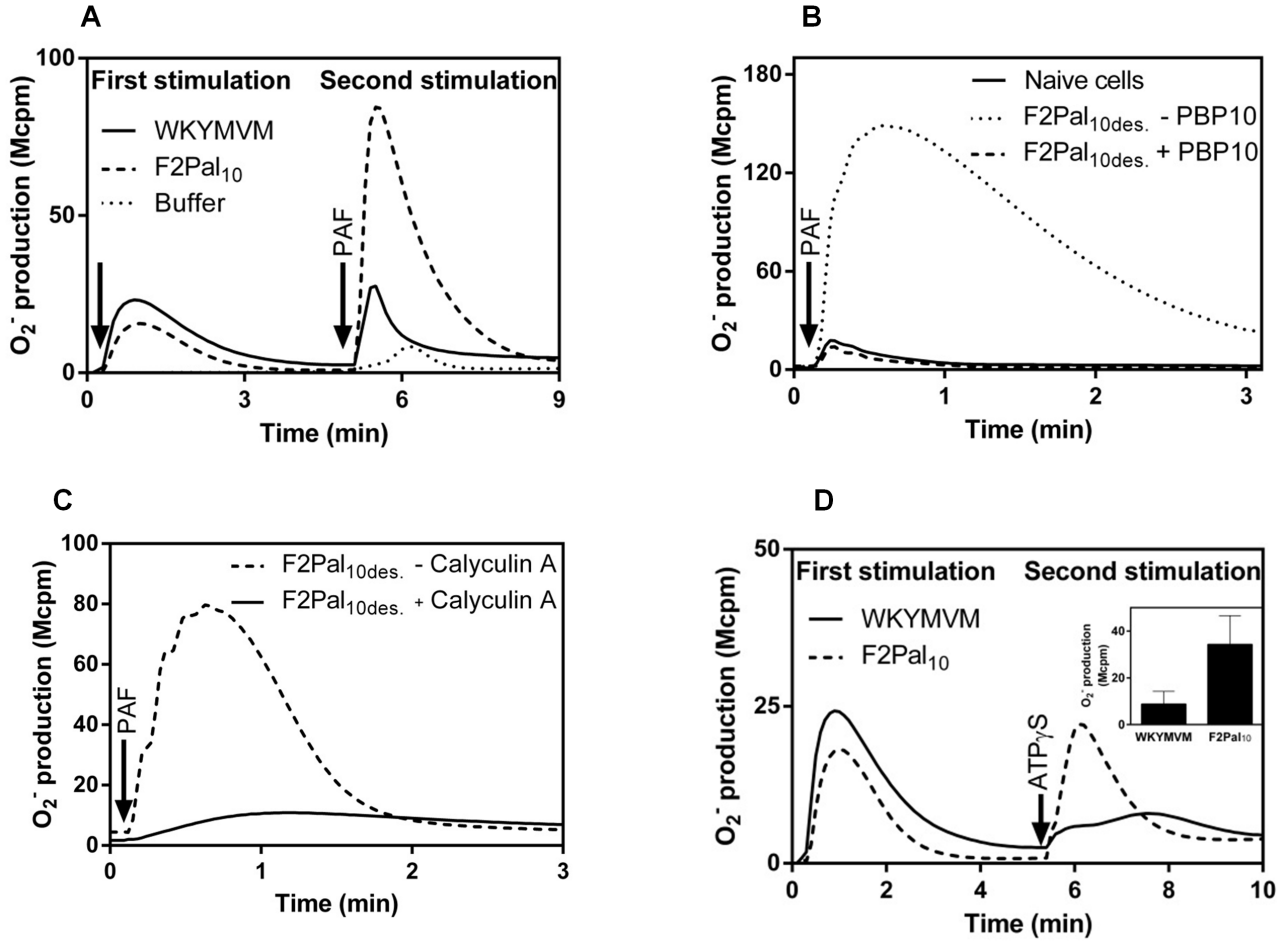
PAF- or ATP-stimulation induces reactivation of desensitized FPR2. **A**. Naïve neutrophils desensitized with F2Pal_10_ (500 nM final concentration) or WKYMVM (40 nM) were primed for subsequent PAF stimulation. A representative experiment is shown and the time point for addition of FPR2 agonists is indicated by the first arrow, the time point for PAF stimulation (100 nM final concentration) with the second arrow. The control PAF response induced in naïve (non-desensitized) neutrophils is shown for comparison (dotted line). Abscissa: Time of study (min); ordinate: Superoxide production (Mcpm). **B.** Naïve neutrophils were first desensitized with F2Pal_10_ (F2Pal_10des_., 500 nM final concentration) and subsequently stimulated with PAF (100 nM final concentration; time point for PAF addition is indicated by an arrow). The FPR2 specific inhibitor PBP10 (1 µM) was added 1 min prior to PAF stimulation (dashed line) and the release of superoxide anions was recorded continuously. The PAF responses induced in naïve cells (solid line), as well as in F2Pal_10_-desensitized cells receiving no PBP10 (dotted line), are shown for comparison. Abscissa: Time of study (min); ordinate: Superoxide production (Mcpm). **C.** Effect of calyculin A on receptor cross-talk induced FPR2 reactivation. Cells desensitized with F2Pal_10_ (F2Pal_10des_.) were incubated with the phosphatase inhibitor calyculin A (60 nM; solid line) or buffer as control (dashed line) for 10 min at 37°C prior to PAF stimulation (100 nM). The release of superoxide anions was recorded continuously. Abscissa: Time of study (min); ordinate: Superoxide production (Mcpm). **D.** Naïve neutrophils were activated with WKYMVM (40 nM final concentration, solid line) or F2Pal_10_ (500 nM final concentration, dashed line) and after the responses had declined the same cells were reactivated with ATPγS (50 µM final concentration; the addition is indicated by an arrow) and the release of superoxide anions was recorded continuously. Abscissa: Time of study (min); ordinate: Superoxide production (Mcpm). Inset: The summary of three independent experiments in which superoxide production was determined using neutrophils desensitized with F2Pal_10_ (500 nM) or WKYMVM (40 nM) when reactivated with ATPγS (50 µM final concentration). The results are given as peak values of superoxide production following addition of ATPγS (mean+SEM; n = 3).

The cellular response induced by FPR agonists is rapidly terminated and the receptors become desensitized to activation by the same agonist and to other agonists that bind to the same receptor. This is true both for WKYMVM and the F2Pal_10_ pepducin, meaning that neutrophils were non-responsive to WKYMVM when desensitized to F2Pal_10_ as well as when the agonist order was reversed (Fig 1B). Our earlier studies have shown that neutrophils desensitized by conventional peptide agonists for FPR1 are primed in their response to ATP and PAF, and this priming relays on a receptor cross-talk mechanism leading to FPR1 reactivation [12], [13]. Neutrophils desensitized by the conventional FPR2 agonist WKYMVM were also primed in their response to PAF (Fig 3A). Similarly, neutrophils desensitized to the F2Pal_10_ pepducin were also largely primed in their response to PAF (Fig 3A). More importantly, the PAF response in FPR2 desensitized neutrophils was inhibited not only by the PAFR specific antagonist WEB2086 (data not shown) but also by FPR2 specific inhibitors when these were added just prior to PAF stimulation (Fig 3B). PAF thus reactivates desensitized FPR2 to an actively signaling state. In agreement with the earlier published data for FPR1 reactivation [13], the cross-talk induced by PAF in FPR2 desensitized cells was inhibited by the phosphatase inhibitor calyculin A (Fig 3C), suggesting that a similar phosphatase sensitive signaling mechanism is involved in reactivation of the two neutrophils FPRs. Very similar results were obtained when PAF was replaced by the P2Y_2_R agonist ATP or the stable analogue ATPγS. The P2Y_2_R agonists can not directly activate the NADPH-oxidase in naïve neutrophils, but they both induced a release of superoxide anions in FPR2 desensitized cells, irrespectively of whether the neutrophils were desensitized by WKYMVM or F2Pal_10_ (shown for ATPγS in Fig 3D) and the reactivation was PBP10 sensitive (data not shown). In summary, the cross-talk potential of FPR2 is induced not only by the conventional receptor specific peptide agonist but also by the unique FPR2 specific pepducin that is proposed to target the receptor directly at the signaling interface.

### The F2Pal_10_ pepducin acts as a full agonist for receptor cross-talk induced FPR2 reactivation

**Figure 4:**
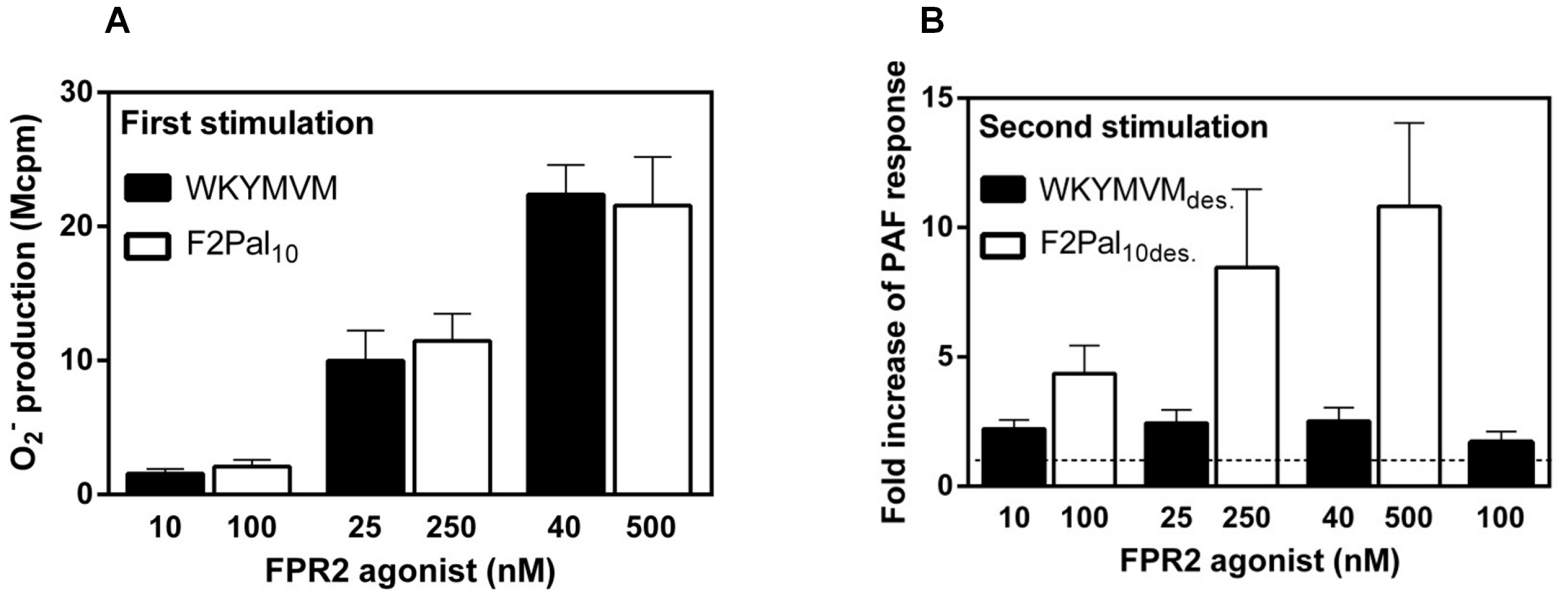
More pronounced reactivation was induced by PAF in neutrophils desensitized by the pepducin F2Pal_10_. **A.** Naïve neutrophils were activated with different concentrations of WKYMVM (10–40 nM; black bars) or F2Pal_10_ (100–500 nM; white bars) followed by a second stimulation with PAF (100 nM final concentration) and the release of superoxide anions was recorded continuously. The results are given as peak values of superoxide production from three separate experiments for the first stimulation with FPR2 agonists (mean+SEM, n = 3). **B.** Neutrophils desensitized with different concentrations of WKYMVM (10–100 nM; WKYMVM_des_., black bars) or F2Pal_10_ (10–500 nM; F2Pal_10des_., white bars) were reactivated by a second stimulation with PAF (100 nM final concentration), after which the superoxide production (peak values) was recorded. The results are given as fold increase (peak values) of the PAF-induced response from FPR2-desensitized cells compared to the PAF response from naïve cells (mean+SEM; n = 3). The naïve PAF response (fold increase = 1) is indicated as a horizontal broken line.

We show that FPR desensitized neutrophils can be reactivated through the cross-talk signals generated by the occupied PAFR and P2Y_2_R, and when it comes to FPR2 both WKYMVM and F2Pal_10_ could be used as desensitizing agents. A difference between the two FPR2 agonists was, however, notable. When the two FPR2 agonists were compared in the cross-talk triggered reactivation system, the PAF-induced FPR2 reactivation was more pronounced in F2Pal_10_ desensitized cells than in WKYMVM desensitized cells (Fig 3). This observation promoted us to examine in more detail whether F2Pal_10_ acts as a full agonist. In order to do this, a reactivation system was used, in which naïve cells were activated (and desensitized) by WKYMVM or F2Pal_10_ at concentrations that induced a comparable sub-maximum response (10 nM WKYMVM≈100 nM F2Pal_10_; 25 nM WKYMVM≈250 nM F2Pal_10_ and; 40 nM WKYMVM≈500 nM F2Pal_10_) (Fig 4A), and these FPR2 desensitized cells were subsequently stimulated with PAF. The reactivation was more pronounced in F2Pal_10_ desensitized cells than in WKYMVM desensitized cells (Fig 4B). The PAF-induced reactivation reached the maximum (10 fold increase) when 500 nM F2Pal_10_ was used. In contrast, the degree of reactivation of WKYMVM desensitized cells was lower (around 2 fold increase) and could not be increased by applying higher WKYMVM concentrations (Fig 4B). Similar results were obtained when WKYMVM was replaced with MMK1, another FPR2 specific peptide agonist, i.e., PAF-induced FPR2 reactivation was less efficient in MMK1- compared to the pepducin-desensitized cells (data not shown). In summary, we show that receptor cross-talk induced FPR2 reactivation was more pronounced in cells desensitized by the pepducin F2Pal_10_ compared to the conventional peptide agonists.

### The pepducin induces typical GPCR signals and oxidase activation in neutrophils

**Figure 5:**
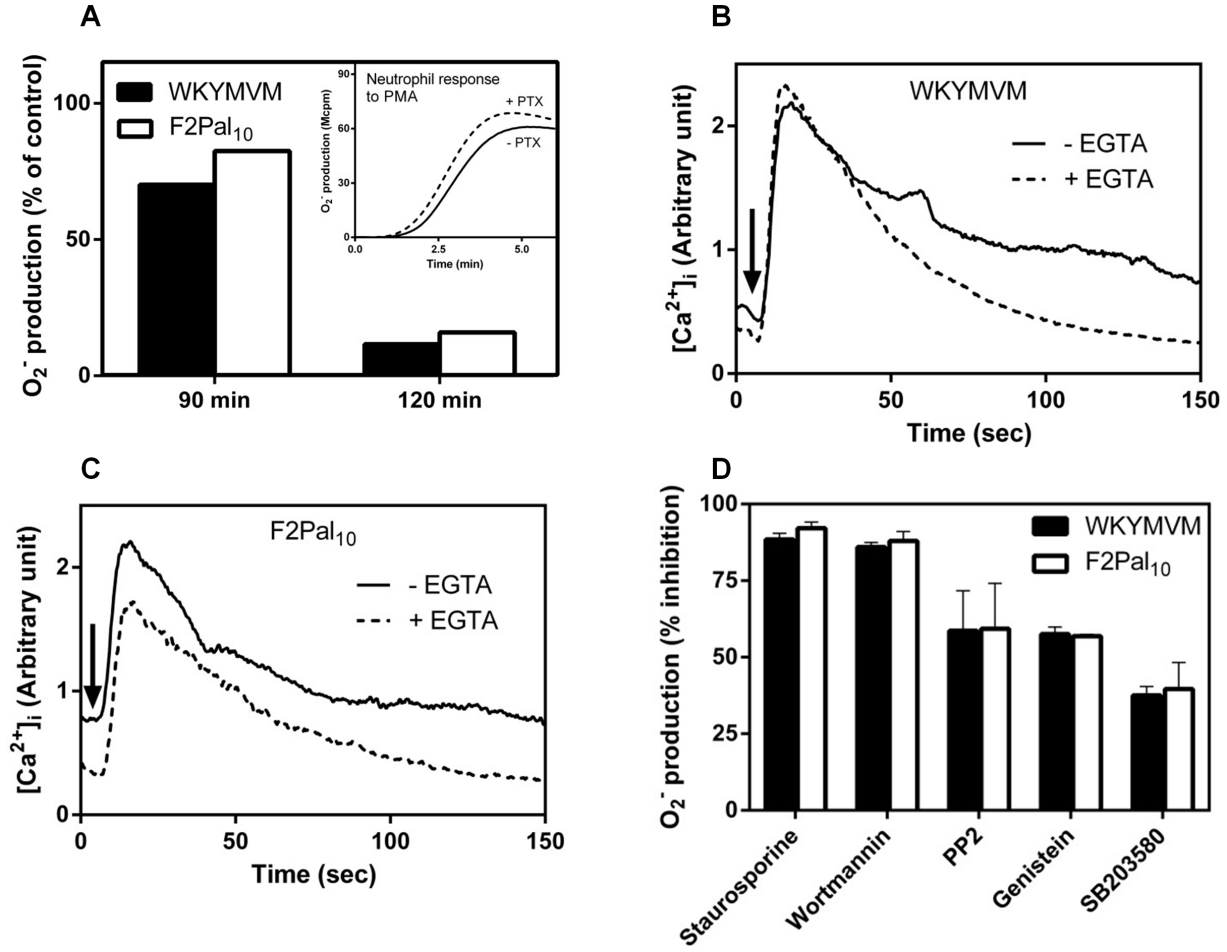
F2Pal_10_ induces pertussis toxin sensitive superoxide production and triggers mobilization of calcium from intracellular stores. A. Primary human neutrophils were treated with pertussis toxin (PTX, 500 ng/ml final concentration) and samples were withdrawn at indicated time points. These cells were activated by WKYMVM (50 nM, black bars) or F2Pal_10_ (500 nM, white bars) and the release of superoxide anions was recorded continuously. The response induced by the peptides was gradually decreased (shown for 90 and 120 min incubation). As a control, the PMA response (5×10^−8^ M final concentration) from control cells (120 min incubation without PTX, solid line in the inset) or treated with PTX for 120 min (dashed line in the inset) is shown. Superoxide production (peak values) from pertussis toxin treated neutrophils was compared to that from non-treated control cells. A representative experiment out of more than five is shown. B and C. Fluo-3-AM/FuraRED labeled neutrophils were incubated without any additive (solid lines) or with EGTA (2 mM; dotted lines). The FPR2 specific peptide WKYMVM (100 nM final concentration; B) or F2Pal_10_ (500 nM; C) was added and the concentration of free cytosolic calcium was monitored by the Fluo-3-AM/FuraRED fluorescence. Traces of representative calcium responses are shown and at least three experiments have been performed. Abscissa, time of study (sec); ordinate, fluorescence (arbitrary unit). D. Primary human neutrophils were incubated with different pharmacological kinase inhibitors (1 µM final concentration) for 15 min at 37°C. Control cells were incubated at the same condition but received no inhibitors. The cells were activated by WKYMVM (50 nM, black bars) or F2Pal_10_ (500 nM, white bars) and the release of superoxide anions was recorded continuously. Data are presented as % inhibition compared to the control response (peak values of superoxide production were used, mean±SEM, n = 3).

The presented data show that the recently described FPR2 pepducin F2Pal_10_ has lower efficacy than WKYMVM in direct receptor-mediated oxidase assembly/activation, however, neutrophils desensitized to the pepducin were more prone to PAF- and ATP-induced reactivation. To gain more insights into the molecular mechanism of action of the pepducin, we used established inhibitor to examine the F2Pal_10_-induced signaling transduction pathways. With respect to the dependency of a heterotrimeric G-protein, we found that the oxidase response induced both by F2Pal_10_ and WKYMVM was largely inhibited by pertussis toxin (Fig 5A). The pertussis toxin treated cells retained their responsiveness to PMA, a neutrophil activator that bypasses the heterotrimeric G-protein (Fig 5A, inset). Apparently and irrespectively of the route of activation, a functional pertussis toxin sensitive Gα_i_-protein downstream of FPR2 is required for both agonists to trigger the production and release of superoxide anions. Further, a rise in the cytosolic concentration of Ca^2+^ was induced in neutrophils by WKYMVM (Fig 5B) as well as by the F2Pal_10_ pepducin (Fig 5C). The rise was evident also when extracellular Ca^2+^ was removed through the addition of EGTA just prior to activation (Fig 5B and C), suggesting that mobilization of Ca^2+^ from intracellular stores is the basis for the rise in cytosolic Ca^2+^. This is in agreement with earlier published data showing that activation with conventional FPR2 agonists results primarily in a mobilization of ions from the storage organelles [31].

To determine the involvement of different kinases in F2Pal_10_- and WKYMVM-induced neutrophil activation, we used a pharmacological approach with several well established kinase inhibitors including SB203580 (a p38MAPK kinase inhibitor), Wortmannin (a PI3K inhibitor), Genistein (a tyrosine kinase inhibitor), PP2 (a Src family kinase inhibitor), and Staurosporine (a protein kinase C inhibitor). The effects of these inhibitors were determined using different concentrations ranging from 20 nM to 1 µM. Very similar degrees of inhibition were obtained for F2Pal_10_- and WKYMVM-induced superoxide release in naïve neutrophils (shown for a 1 µM concentration of the inhibitors in Fig 5D). Taken together, these data show that with respect to neutrophil activation, F2Pal_10_ and the conventional FPR2 agonist possess many similarities at the signaling level.

**Figure 6:**
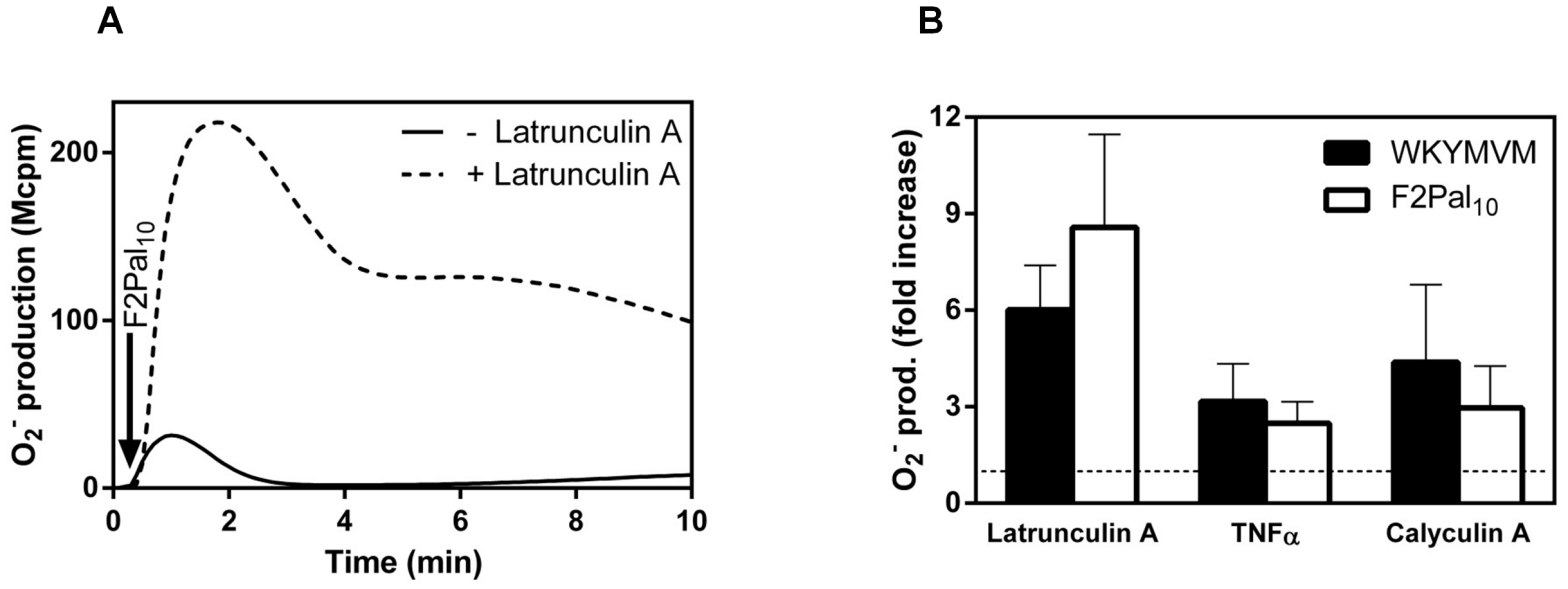
The neutrophil response to the pepducin F2Pal_10_ is primed by latrunculin A, TNFα and calyculin A. WKYMVM (50 nM final concentration) or F2Pal_10_ (500 nM final concentration) were added to control (non-primed) or treated neutrophils and the release of superoxide anions was recorded continuously. **A.** A representative experiment of an F2Pal_10_ induced response in control (solid line) and latrunculin A treated neutrophils (dashed line) is shown. The time point for addition of F2Pal_10_ is indicated by an arrow. Abscissa: Time of study (min); ordinate: Superoxide production (Mcpm). **B.** The priming effects of latrunculin A, TNFα and Calyculin A on the WKYMVM (50 nM final concentration, black bars) or F2Pal_10_ (500 nM final concentration, white bars) are summarized as fold increase (peak values of the superoxide production) when compared to the response in non-treated cells (mean ± SEM; n = 3). A non-priming cut-off line (fold increase = 1) is included as a horizontal broken line in the figure.

When the response induced by FPR agonists in neutrophils is terminated, the occupied receptors become desensitized through an interaction between the receptor/ligand complex and the actin cytoskeleton [32], [33], [34]. Accordingly, the neutrophil response to F2Pal_10_ was dramatically increased and prolonged in the presence of latrunculin A, a drug that sequesters actin monomers and prevents actin polymerization, suggesting an involvement of the actin cytoskeleton in F2Pal_10_ signaling (Fig 6A). The oxidase activation induced by F2Pal_10_ was also examined in cells presented in different cellular states, i.e., naïve or primed. The primed state can be induced by many different priming agents such as LPS, TNFα and the phosphatase inhibitor calyculin A [35], [36], [37]. Accordingly, neutrophils primed with TNFα or calyculin A produced significantly more superoxide than non-primed control cells when WKYMVM was used to induce the response (Fig 6B). Very similar priming effects were obtained when F2Pal_10_ was used as the triggering agonist instead of WKYMVM (Fig 6B), suggesting that the F2Pal_10_ response is also largely dependent on the cellular state examined.

Taken together, these data show that the pepducin F2Pal_10_ and the conventional peptide agonist WKYMVM utilize similar intracellular signaling pathways and trigger very similar patterns of oxidase activation in neutrophils.

## Discussion

### Mechanisms of GPCR activation

The generally accepted mechanism for how GPCRs are activated anticipates a conventional agonist that binds to a defined binding pocket localized in receptor domains available for interaction on the cell surface, and the occupied receptor is then transferred from a non-signaling state to a stabilized and active signaling state. This two-state model is today regarded as an over-simplification of how receptor signaling is regulated [38], [39], [40]. New mechanisms have been introduced through the use of molecules supposed to regulate receptor functions from the cytosolic side of the plasma membrane [41], [42]. In accordance with this, pepducins containing an amino acid sequence derived from one of the cytoplasmic parts of a GPCR, activate a given receptor by mechanisms suggested to differ from those used by conventional peptide agonists [17]. The precise mechanism for how the membrane-binding and transport steps of pepducins are achieved/regulated have not been worked out at the molecular level, but once inside the cell membrane, the generally accepted model stipulates that the peptide moiety of the pepducin interacts with targeted receptor-region(s) exposed on the inner leaflet of the membrane. The suggested mechanisms for activation include, i) a direct stabilization of the receptor/G-protein complex possibly by mimicking a dimerization, and ii) direct binding to the receptor that allosterically stabilizes the receptor in a specific conformation that facilitates G-protein interaction/activation [17]. It is very hard to understand how pepducins with amino acid sequences identical to a cytosolic part of a GPCR could activate its specific receptor through an interaction with cytosolic parts of this receptor [14], [17], but at the same time, the alternative mode of action at hand (that pepducins function as conventional agonists with selectivity/specificity for the receptor from which the amino acid sequence is derived) is as spectacular. With respect to the FPR2 pepducin, one criterion fulfilled for other GPCR pepducins is not met, namely insensitivity to extracellular receptor antagonists; this suggests that there might be alternative mechanisms of action for this pepducin [14]. Furthermore, the competition in binding between the FPR2 pepducin and the conventional FPR2 agonist WKYMVM leaves the possibility that F2Pal_10_ may act from the extracellular side of the receptor. Irrespective of the precise mechanism, it is of importance to notice that there are no sequence similarities at the amino acid level between the different pepducins reported, and a pepducin must contain a fatty acid that permits the molecule to pass the plasma membrane. Moreover, the pepducin-induced responses are receptor specific, meaning that the receptor from which the peptide sequence is derived has to be expressed in the responding cell [14], [17].

### Neutrophil activation by FPR2 agonists

The F2Pal_10_ pepducin derived from the third intracellular loop of FPR2 activates neutrophils through FPR2 but not the closely related FPR1, despite the fact that the two FPRs have sequences in the third intracellular loops that differ in only two amino acid positions. We have previously shown that the agonistic effect of the F2Pal pepducin relies on the fatty acid and is affected by the length of the fatty acid anchor as well as the length and charge of the peptide [14]. Neutrophil activation patterns induced by F2Pal_10_ and WKYMVM are very similar and the peak in radical production is reached very rapidly upon stimulation (after around one minute) and once signaling has terminated the receptor is homologously desensitized. In addition, neutrophils activated by the pepducin were desensitized and non-responsive also to conventional peptide receptor agonists. Although the activation of naïve neutrophils by the F2Pal_10_ pepducin appears very similar to that induced by the conventional FPR2 agonist WKYMVM, the maximum response (measured as the peak of the response) induced by the F2Pal_10_ pepducin is only around 70% of the response induced by WKYMVM. Receptor agonists could be classified in relation to the activity induced; a full agonist for a given receptor being one that gives a full response whereas a partial agonist for the same receptor should induce a response with a partial efficacy (sub-maximum). According to this definition, F2Pal_10_ should be classified as a partial FPR2 agonist when it comes to direct receptor-mediated activation of naïve neutrophils. The relative potency of the conventional peptide agonist WKYMVM and F2Pal_10_ was, however, reversed regarding the cross-talk reactivation induced by PAF and ATP. In cross-talk reactivation, the F2Pal_10_ pepducin is a full agonist and WKYMVM is a partial agonist. Thus, a pepducin can function both as a full and as a partial agonist, depending on the precise functional response used to determine the type of agonism.

### Receptor cross-talk induced FPR2 reactivation

We have previously shown that the signals generated by the occupied PAFR and P2Y_2_R reactivate FPR1 desensitized with conventional FPR1 agonists [12], [13], a novel mechanism for amplification of neutrophil production of reactive oxygen species. The results presented in this study show that FPR2 was sensitive to the same reactivation signals generated by PAFR and P2Y_2_R. Our data reveal, however, a significant difference between the desensitizing agents used when it comes to reactivation of desensitized FPR2, i.e., the reactivation induced by the PAFR and by P2Y_2_R was much more pronounced in neutrophils desensitized with the F2Pal_10_ pepducin compared to those desensitized to the conventional agonist WKYMVM. Although we have made several attempts to gain knowledge about the molecular mechanisms that underlie receptor desensitization and reactivation *per se* [12], [13], we can at present only speculate on the regulatory mechanisms that are operating. A full understanding of the mechanisms behind the differences between the pepducin and the conventional agonist in relation to receptor reactivation is prohibited by the general lack in basic knowledge regarding termination of signaling in agonist occupied FPRs. Moreover, increased understanding of the precise mechanism by which pepducins interact with their target receptors would undoubtedly help the development of models explaining how certain GPCRs communicate with each other during cross-talk induced receptor reactivation.

### Concluding remarks

We show that the F2Pal_10_ pepducin derived from the third intracellular loop of FPR2 triggers a functional response in neutrophils through FPR2, and the activation pattern is very similar to that induced by the conventional FPR2 peptide agonist WKYMVM. The functional dualism of the F2Pal_10_ pepducin, being a partial agonist for direct activation of FPR2 and a full agonist for PAF- and ATP-induced reactivation of desensitized FPR2, represents a type of functional selectivity, a phenomenon recently described for other pepducins exhibiting biased signaling of discrete cellular functions [17], [41]. Earlier reports have stated that pepducins permeate the plasma membrane and may act from the cytosolic side of the receptor, and the cellular effects induced by a particular pepducin apparently require the presence of the receptor from which its peptide sequence is derived [17], [41]. More studies are required to elucidate the precise mechanism of action of pepducins in general, and how F2Pal_10_ mediates a pronounced amplification of superoxide production in neutrophils achieved through the described receptor cross-talk mechanism.

## Supporting Information

Figure S1 **The FPR2 pepducin F2Pal_10_ inhibits binding of the Cy5-WKYMVM to human neutrophils.** Human neutrophils were incubated with the fluorescently labeled Cy5-WKYMVM peptide (1 nM final concentration) in the absence (1 hour contol, solid line) or presence of an excess of non-labeled WKYMVM (100 nM final concentration, dashed line) or F2Pal_10_ (1 µM final concentration, dotted line) on ice. The cells were incubated for 1 or 2 hours before analysis by flow cytometry. One representative histogram for Cy5-WKYMVM fluorescence after a 1 hour incubation period is shown. The inset shows the inhibition of Cy5-WKYMVM binding upon incubation with non-labeled peptides for 1 hour and 2 hours, respectively.
(TIF)

Figure S2 **The presence of a saturating amount of F2Pal_10_ reduces the neutrophil production O_2_^−^ in response of WKYMVM.** Neutrophils were activated by WKYMVM and F2Pal_10_ through an addition of the two agonists separately (1 µM WKYMVM; black bar, and 1 µM F2Pal_10_; white bar) or the two together (1 µM each; grey bar), and the release of superoxide anions was recorded continuously. The peak values of superoxide production were determined and expressed in arbitrary units (mean±SEM, n = 3).
(TIF)

## References

[1] Fu H, Karlsson J, Bylund J, Movitz C, Karlsson A, et al (2006) Ligand recognition and activation of formyl peptide receptors in neutrophils. J Leukoc Biol 79: 247–256. 16365159 10.1189/jlb.0905498

[2] Ye RD, Boulay F, Wang JM, Dahlgren C, Gerard C, et al (2009) International Union of Basic and Clinical Pharmacology. LXXIII. Nomenclature for the formyl peptide receptor (FPR) family. Pharmacol Rev 61: 119–161. 19498085 10.1124/pr.109.001578 PMC2745437

[3] Murphy PM (1994) The molecular biology of leukocyte chemoattractant receptors. Annu Rev Immunol 12: 593–633. 8011292 10.1146/annurev.iy.12.040194.003113

[4] Ye RD, Boulay F (1997) Structure and function of leukocyte chemoattractant receptors. Adv Pharmacol 39: 221–289. 9160117 10.1016/s1054-3589(08)60073-3

[5] Eglen R, Reisine T (2011) GPCRs revisted: New Insights lead to novel drugs. Pharmaceuticals 4: 244–272.

[6] Kolaczkowska E, Kubes P (2013) Neutrophil recruitment and function in health and inflammation. Nat Rev Immunol 13: 159–175. 23435331 10.1038/nri3399

[7] Sadik CD, Kim ND, Luster AD (2011) Neutrophils cascading their way to inflammation. Trends Immunol 32: 452–460. 21839682 10.1016/j.it.2011.06.008 PMC3470857

[8] Magalhaes AC, Dunn H, Ferguson SS (2012) Regulation of GPCR activity, trafficking and localization by GPCR-interacting proteins. Br J Pharmacol 165: 1717–1736. 21699508 10.1111/j.1476-5381.2011.01552.x PMC3372825

[9] Audet M, Bouvier M (2012) Restructuring G-protein- coupled receptor activation. Cell 151: 14–23. 23021212 10.1016/j.cell.2012.09.003

[10] Hendriks-Balk MC, Peters SL, Michel MC, Alewijnse AE (2008) Regulation of G protein-coupled receptor signalling: focus on the cardiovascular system and regulator of G protein signalling proteins. Eur J Pharmacol 585: 278–291. 18410914 10.1016/j.ejphar.2008.02.088

[11] Giniatullin R, Nistri A, Yakel JL (2005) Desensitization of nicotinic ACh receptors: shaping cholinergic signaling. Trends Neurosci 28: 371–378. 15979501 10.1016/j.tins.2005.04.009

[12] Önnheim K, Christenson K, Gabl M, Burbiel JC, Muller CE, et al (2014) A novel receptor cross-talk between the ATP receptor P2Y and formyl peptide receptors reactivates desensitized neutrophils to produce superoxide. Exp Cell Res 323: 209–217. 24491917 10.1016/j.yexcr.2014.01.023

[13] Forsman H, Önnheim K, Andreasson E, Christenson K, Karlsson A, et al (2013) Reactivation of desensitized formyl peptide receptors by platelet activating factor: a novel receptor cross talk mechanism regulating neutrophil superoxide anion production. PLoS One 8: e60169. 23555913 10.1371/journal.pone.0060169 PMC3610682

[14] Forsman H, Bylund J, Oprea TI, Karlsson A, Boulay F, et al (2013) The leukocyte chemotactic receptor FPR2, but not the closely related FPR1, is sensitive to cell-penetrating pepducins with amino acid sequences descending from the third intracellular receptor loop. Biochim Biophys Acta 1833: 1914–1923. 23562731 10.1016/j.bbamcr.2013.03.026

[15] Bylund J, Gabl M, Winther M, Onnheim K, Dahlgren C, et al (2014) Turning Chemoattractant Receptors On and Off with Conventional Ligands and Allosteric Modulators: Recent advances in formyl peptide receptor signaling and regulation. Inflamm & Cell Signal 1: 24–38.

[16] Covic L, Gresser AL, Talavera J, Swift S, Kuliopulos A (2002) Activation and inhibition of G protein-coupled receptors by cell-penetrating membrane-tethered peptides. Proc Natl Acad Sci U S A 99: 643–648. 11805322 10.1073/pnas.022460899 PMC117359

[17] O'Callaghan K, Kuliopulos A, Covic L (2012) Turning receptors on and off with intracellular pepducins: new insights into G-protein-coupled receptor drug development. J Biol Chem 287: 12787–12796. 22374997 10.1074/jbc.R112.355461 PMC3339939

[18] Tressel SL, Koukos G, Tchernychev B, Jacques SL, Covic L, et al (2011) Pharmacology, biodistribution, and efficacy of GPCR-based pepducins in disease models. Methods Mol Biol 683: 259–275. 21053136 10.1007/978-1-60761-919-2_19 PMC3780409

[19] Jamieson T, Clarke M, Steele CW, Samuel MS, Neumann J, et al (2012) Inhibition of CXCR2 profoundly suppresses inflammation-driven and spontaneous tumorigenesis. J Clin Invest 122: 3127–3144. 22922255 10.1172/JCI61067 PMC3428079

[20] Heit B, Tavener S, Raharjo E, Kubes P (2002) An intracellular signaling hierarchy determines direction of migration in opposing chemotactic gradients. J Cell Biol 159: 91–102. 12370241 10.1083/jcb.200202114 PMC2173486

[21] Forsman H, Andreasson E, Karlsson J, Boulay F, Rabiet MJ, et al (2012) Structural characterization and inhibitory profile of formyl peptide receptor 2 selective peptides descending from a PIP2-binding domain of gelsolin. J Immunol 189: 629–637. 22706076 10.4049/jimmunol.1101616

[22] Kemp PA, Sugar RA, Jackson AD (2004) Nucleotide-mediated mucin secretion from differentiated human bronchial epithelial cells. Am J Respir Cell Mol Biol 31: 446–455. 15231488 10.1165/rcmb.2003-0211OC

[23] Meghani P (2002) The design of P2Y2 antagonists for the treatment of inflammatory diseases. Abstracts of papers:American Chemical Society.

[24] Kindon N, Meghani P.,Thom S (1998) Novel compounds. WO1998054180.

[25] Boyum A, Lovhaug D, Tresland L, Nordlie EM (1991) Separation of leucocytes: improved cell purity by fine adjustments of gradient medium density and osmolality. Scand J Immunol 34: 697–712. 1749920 10.1111/j.1365-3083.1991.tb01594.x

[26] Bylund J, Bjornsdottir H, Sundqvist M, Karlsson A, Dahlgren C (2014) Measurement of respiratory burst products, released or retained, during activation of professional phagocytes. Methods Mol Biol 1124: 321–338. 24504962 10.1007/978-1-62703-845-4_21

[27] Dahlgren C, Karlsson A, Bylund J (2007) Measurement of respiratory burst products generated by professional phagocytes. Methods Mol Biol 412: 349–363. 18453123 10.1007/978-1-59745-467-4_23

[28] Dahlgren C, Christophe T, Boulay F, Madianos PN, Rabiet MJ, et al (2000) The synthetic chemoattractant Trp-Lys-Tyr-Met-Val-DMet activates neutrophils preferentially through the lipoxin A(4) receptor. Blood 95: 1810–1818. 10688842

[29] Karlsson J, Bylund J, Movitz C, Bjorkman L, Forsman H, et al (2010) A methodological approach to studies of desensitization of the formyl peptide receptor: Role of the read out system, reactive oxygen species and the specific agonist used to trigger neutrophils. J Immunol Methods 352: 45–53. 19891970 10.1016/j.jim.2009.10.011

[30] Lee HY, Kim SD, Shim JW, Kim HJ, Kwon JY, et al (2010) Activation of human monocytes by a formyl peptide receptor 2-derived pepducin. FEBS letters 584: 4102–4108. 20804753 10.1016/j.febslet.2010.08.036

[31] Karlsson J, Stenfeldt AL, Rabiet MJ, Bylund J, Forsman HF, et al (2009) The FPR2-specific ligand MMK-1 activates the neutrophil NADPH-oxidase, but triggers no unique pathway for opening of plasma membrane calcium channels. Cell Calcium 45: 431–438. 19282028 10.1016/j.ceca.2009.02.002

[32] Bylund J, Bjorstad A, Granfeldt D, Karlsson A, Woschnagg C, et al (2003) Reactivation of formyl peptide receptors triggers the neutrophil NADPH-oxidase but not a transient rise in intracellular calcium. J Biol Chem 278: 30578–30586. 12773548 10.1074/jbc.M209202200

[33] Jesaitis AJ, Klotz KN (1993) Cytoskeletal regulation of chemotactic receptors: molecular complexation of N-formyl peptide receptors with G proteins and actin. Eur J Haematol 51: 288–293. 8282090 10.1111/j.1600-0609.1993.tb01610.x

[34] Jesaitis AJ, Tolley JO, Painter RG, Sklar LA, Cochrane CG (1985) Membrane-cytoskeleton interactions and the regulation of chemotactic peptide-induced activation of human granulocytes: the effects of dihydrocytochalasin B. J Cell Biochem 27: 241–253. 3838753 10.1002/jcb.240270306

[35] Almkvist J, Faldt J, Dahlgren C, Leffler H, Karlsson A (2001) Lipopolysaccharide-induced gelatinase granule mobilization primes neutrophils for activation by galectin-3 and formylmethionyl-Leu-Phe. Infect Immun 69: 832–837. 11159975 10.1128/IAI.69.2.832-837.2001 PMC97959

[36] Bylund J, Karlsson A, Boulay F, Dahlgren C (2002) Lipopolysaccharide-induced granule mobilization and priming of the neutrophil response to *Helicobacter pylori* peptide Hp(2–20), which activates formyl peptide receptor-like 1. Infect Immun 70: 2908–2914. 12010979 10.1128/IAI.70.6.2908-2914.2002 PMC127963

[37] Karlsson A, Follin P, Leffler H, Dahlgren C (1998) Galectin-3 activates the NADPH-oxidase in exudated but not peripheral blood neutrophils. Blood 91: 3430–3438. 9558402

[38] Manglik A, Kobilka B (2014) The role of protein dynamics in GPCR function: insights from the betaAR and rhodopsin. Curr Opin Cell Biol 27C: 136–143. 10.1016/j.ceb.2014.01.008 PMC3986065 24534489

[39] Seifert R (2013) Functional selectivity of G-protein-coupled receptors: from recombinant systems to native human cells. Biochem Pharmacol 86: 853–861. 23933388 10.1016/j.bcp.2013.07.029

[40] Stallaert W, Christopoulos A, Bouvier M (2011) Ligand functional selectivity and quantitative pharmacology at G protein-coupled receptors. Expert Opin Drug Discov 6: 811–825. 22651124 10.1517/17460441.2011.586691

[41] Quoyer J, Janz JM, Luo J, Ren Y, Armando S, et al (2013) Pepducin targeting the C-X-C chemokine receptor type 4 acts as a biased agonist favoring activation of the inhibitory G protein. Proc Natl Acad Sci U S A 110: E5088–5097. 24309376 10.1073/pnas.1312515110 PMC3876208

[42] Shukla AK (2014) Biasing GPCR signaling from inside. Sci Signal 7: 1–2. 10.1126/scisignal.2005021 24473194

